# Maternal intranasal immunization with HTLV-1 Env induces Env specific antibody responses in breast milk

**DOI:** 10.1186/1742-4690-12-S1-P88

**Published:** 2015-08-28

**Authors:** Tadaki Suzuki

**Affiliations:** 1 National Institute of Health and Nutrition, Japan

## 

Human T cell lymphotropic virus type-1 (HTLV-1) is the etiologic agent of Adult T cell Leukemia and Lymphoma (ATLL) and the neurological disorder HTLV-I-associated myelopathy/tropical spastic paraparesis (HAM/TSP). The virus prevalent worldwide with foci of high prevalence. However, effective vaccines or drugs against HTLV-1 infection are not available. Because of the severity of the diseases and the absence of a satisfactory treatment, there is indeed an urgent need for an efficient vaccine against HTLV-1 in endemic regions. HTLV-1 is associated with persistent and lifelong infections and a low incidence of lymphomas within its hosts, and can be spread through the contact with bodily fluids containing infected cells, most often from mother to offspring through breast milk. These previous observations suggest that HTLV-1 has common features in infection and protective immune mechanisms with Bovine Leukemia virus (BLV). Imm une protection of newborns by vaccination is difficult to achieve since there is not enough time to mount an immune response before exposure to the virus. Interestingly, previous study revealed that antibodies from the maternal colostrum protect from BLV infection. On the basis of the above reports, we have designed a vaccination strategy mediating transfer of neutralizing antibodies from the mother to the offspring during pregnancy and / or lactation. In the present study, we investigated the effectiveness of intranasal vaccine using HTLV-1 Env protein as an antigen in combination with a synthetic double-stranded RNA analog for the induction of specific antibody in serum and breast milk in mother mice. Full-length Env protein was synthesized using the wheat germ cell-free protein production system. Intranasal administration for mother mice with the Env protein and the synthetic double-stranded RNA adjuvant, polyriboinosinic-polyribocytidylic acid [poly(I:C)] generated significant Env-specific systemic and mucosal immune responses as evidenced by the prominent induction of serum and milk antibodies. Our results provide the first evidence that intranasal co-administration of HTLV-1 Env protein with the poly(I:C) adjuvant augments the viral-specific immunity against HTLV-1.

